# Body weight, organ development and jejunal histomorphology in broiler breeder pullets fed n-3 fatty acids enriched diets from hatch through to 22 weeks of age

**DOI:** 10.1016/j.psj.2021.101514

**Published:** 2021-10-08

**Authors:** Aizwarya Thanabalan, Elijah G. Kiarie

**Affiliations:** Department of Animal Biosciences, University of Guelph, Guelph, ON N1G 2W1, Canada

**Keywords:** broiler breeder pullet development, organ weight, omega-3 fatty acids, body weight uniformity

## Abstract

Dietary long chain polyunsaturated n-3 fatty acids (**n-3 FA**) may be beneficial to broiler breeder (**BB**) development. Therefore, the effects of feeding sources of docosahexaenoic acid (**DHA**) and α-linolenic acid (**ALA**) from hatch through to 22 weeks of age (**woa**) on growth, organ weight, and jejunal histomorphology were investigated. A total of 588-day-old Ross × Ross 708 BB were reared on one of 3 diets: 1) control, corn-soybean meal diet, 2) Control + 1% microalgae (**DMA**, *Aurantichytrium limacinum*), as a source of DHA and 3) Control + 2.50% co-extruded full fat flaxseed and pulse mixture (**FFF**, 1:1 wt/wt), as a source of ALA. Diets DMA and FFF had similar total n-3 and n-6: n-3 ratio. Diets were allocated to floor pens (28 birds/pen) to give 9 or 6 replicates per diet for control or DMA and FFF, respectively and fed according to breeder curve in 3 phases: starter (0–4 woa), grower (5–19 woa), and pre-breeder (20–22 woa). Individual body weight (**BW**) was taken weekly and 6 birds/pen necropsied at 5 and 12 woa for gastrointestinal, spleen, bursa, and liver weight and samples for jejunal histomorphology. There was no (*P* > 0.05) diet effect on growth by 20 woa. With exception of 5 woa, pullets fed DMA showed (*P* < 0.001) lower BW coefficient of variation (**C.V**.) than pullets fed control between 2 and 7 woa. However, pullets fed DMA had higher BW CV at 20 woa than birds fed either control or FFF. At 5 woa, birds fed DMA had taller (*P* ≤ 0.01) villi and deeper crypt than birds fed either control or FFF but VH or CD were similar (*P* > 0.05) between CON and FFF pullets. At 12 woa, birds fed FFF had taller VH than birds fed control diet but similar (*P* > 0.05) to that of birds fed DMA. Therefore, different responses to sources of omega-3 FA may implicate other components, however, the BW uniformity and intestinal histomorphology responses suggested benefits of feeding omega-3 FA during rearing.

## INTRODUCTION

Broiler breeders (**BB**) have been genetically selected for commercially favorable traits such as rapid and efficient growth and a successful lifetime egg production (Richards et al., 2010). The metabolic needs resulting from these features are contradictory as broiler chickens require high feed intake, yet excess body weight in BB leads to low egg production and egg size variability. Consequently, BB are feed restricted, starting from the rearing phase to control body weight at sexual maturity (Zuidhof et al., 2015). Restricted feeding regimens produce stress responses, potentially leaving birds immunocompromised (Hocking et al., 1996, De Jong et al., 2002). Broiler breeder pullets are also exposed to other stressors associated with routine rearing practices, including vaccination and handling that can have an immunocompromising impact. Whether these stressors occur singly or simultaneously, they have the potential to stimulate the stress response, impair immunity, and negatively affect bird growth and well-being (Klasing, 2007). The intestinal tract, the immune system, and the integument (skin and feathers) undergo a lot of growth and development in the first 6 wk of pullet life. Pullets that have higher levels of stress during this growth spurt are more likely to have poor uniformity, which can negatively affect reproductive performance (Leeson and Summers, 2005)

In recent years omega-3 fatty acids inclusion in poultry diets have attracted interest due to their diverse roles in organ development and immune response modulation (Calder, 2013). Long-chain polyunsaturated n-3 fatty acids (**n-3 FA**) such as docosahexaenoic acid (22:6 n-3; **DHA**) and eicosapentaenoic acid (20:5 n-3; **EPA**) are critical for optimal cell, tissue, and organ development (Koppenol et al., 2014). These fatty acids are needed for prenatal and postnatal development due to their vital roles in the synthesis of structural lipids (Cherian, 2011, 2015; Mennitti et al., 2015; Thanabalan and Kiarie, 2021). Dietary omega-3 fatty acids can be supplied as the parent n-3 FA α-linolenic acid (**ALA**) through plant sources such as oilseeds or DHA supplying marine sources such as fish oil or algae (Swiatkiewicz et al., 2015). However, little research has been conducted on the effects manipulating dietary fatty acids composition during BB rearing. This study aimed to examine the effects of feeding 2 n-3 FA sources in BB pullets rearing feeding program on growth, organ development and intestinal histomorphology through to 22 weeks of age (**woa**). It was hypothesized that inclusion of either n-3 FA sources would improve body weight uniformity and organ development.

## MATERIALS AND METHODS

The experimental protocol was approved by the University of Guelph Animal Care Committee, and birds were cared for in accordance with the Canadian Council on Animal Care guidelines (CCAC, 2009).

### Birds and Housing

A total of 588-day-old broiler breeder pullets (Ross 708) were procured from Aviagen (Aviagen Inc., Huntsville, AL) through the Ontario Broiler Hatching Egg and Chick Commission. Chicks were beak treated using an infrared beam at the hatchery. All birds were weighed, to ensure uniform bodyweight between pens, wing tagged and placed in 21 floor pens (28 BB/pen) housed in 2 environmentally controlled rooms at Arkell Poultry Research Station, University of Guelph (Guelph, ON). The pens (2.36 m wide × 1.83 m deep) were bedded with fresh wood shavings and equipped with a trough feeder and 6 nipple drinkers. The temperature was set at 32°C on d 0 and decreased by 2°C weekly to 21°C. From d 0 to 4, birds were given 23L:1D (20 lux) then switched to 12L:12D (20 lux) until 2 woa. Birds were exposed to 8L:16D (12 lux) up to 3 woa after which lighting was switched to 14L:10D (20 lux) until the end of the experiment. Birds were vaccinated for Marek disease and coccidiosis at hatchery. Birds were vaccinated at 5 (spray), 10 (spray), and 16 (intramuscularly) woa for Newcastle and infectious bronchitis. Birds were also vaccinated with Infectious Laryngotracheitis Vectormune FP-LT-AE (wing web) at 8 woa.

### Diets

Three diets were 1) control, formulated to meet specifications (Aviagen, 2013), 2) control + 1% microalgae (**DMA**, *Aurantichytrium limacinum*) as a source of DHA (Ao et al., 2015), (Alltech Canada, Guelph ON, Canada) and 3) control + 2.50% co-extruded full fat flaxseed and pulse mixture (**FFF**, 1:1 wt/wt) as a source of alpha-linolenic acid (linPRO, O & T Farms Ltd., Regina, SK, Canada). Fatty acid enriched diets were formulated such that the ratio of total n-3 to n-6 fatty acids were similar between the experimental diets, accounting for some limitations due to meeting nutrient requirements, and overall nutrients remained constant between all 3 diets (Table 1). Diet phases were formulated and changed as per the broiler breeder management guide: Starter (0–4 woa), grower (5–19 woa), and prebreeder (20–22 woa; Aviagen, 2013).

**Table 1:** Ingredient and calculated nutrient composition of experimental diets on an as fed basis^1^.

Item	Starter			Grower			Pre-breeder		
Ingredient, %	Control	DMA	FFF	Control	DMA	FFF	Control	DMA	FFF
Wheat	35.7	34.5	32.8	20.6	18.1	19.9	20.6	16.4	18.6
Corn	30.0	30.9	31.8	30.0	30.0	30.0	30.0	30.0	30.0
Soybean meal	26.3	26.2	25.6	17.1	17.0	16.1	11.5	11.5	10.6
DMA^2^	-	1.00	-	-	1.00	-	-	1.00	-
FFF^3^	-	-	2.32	-	-	2.32	-	-	2.32
Corn oil	0.49	-	-	0.50	0.16	0.00	0.50	0.30	0.10
Wheat middlings	-	-	-	15.0	15.0	15.0	15.0	15.0	15.0
Barley	-	-	-	8.88	10.7	8.48	13.7	17.0	14.4
Oat hulls	-	-	-	3.00	3.00	3.00	3.00	3.00	3.00
Corn starch	2.00	2.00	2.00	-	-	-	-	-	-
Monocalcium phosphate	1.98	1.99	1.99	1.89	1.88	1.89	1.54	1.52	1.53
Limestone fine	1.42	1.41	1.40	1.19	1.19	1.19	2.16	2.16	2.15
Vitamin and trace mineral premix^4^	1.00	1.00	1.00	1.00	1.00	1.00	1.00	1.00	1.00
Salt	0.26	0.26	0.26	0.29	0.29	0.29	0.27	0.27	0.27
DL-Methionine, 99%	0.23	0.22	0.23	0.16	0.16	0.17	0.18	0.18	0.18
L-Lysine HCl, 78%	0.20	0.20	0.20	0.07	0.07	0.08	0.15	0.14	0.15
L-Threonine, 98%	0.09	0.10	0.10	0.08	0.08	0.08	0.10	0.10	0.11
L-Tryptophan, 98%	-	-	-	-	-	-	0.01	0.01	0.01
Multi-carbohydrase supplement^5^	0.05	0.05	0.05	0.05	0.05	0.05	0.05	0.05	0.05
Ethoxyquin^6^	0.02	0.02	0.02	0.02	0.02	0.02	0.02	0.02	0.02
Sodium bicarbonate	0.19	0.19	0.19	0.15	0.15	0.15	0.18	0.18	0.18
Calculated nutrients									
AME, kcal/kg	2,800	2,800	2,800	2,800	2,800	2,800	2,800	2,800	2,800
Crude protein, %	19.0	19.0	19.0	16.5	16.5	16.5	14.5	14.5	14.5
Crude fat, %	2.47	2.69	2.48	2.98	3.62	3.43	3.06	3.66	3.20
SID Lysine, %	0.95	0.95	0.95	0.61	0.61	0.61	0.54	0.54	0.54
SID Methionine, %	0.48	0.48	0.48	0.38	0.38	0.38	0.34	0.34	0.34
SID Met + Cys, %	0.75	0.75	0.75	0.59	0.59	0.59	0.52	0.52	0.52
Calcium, %	0.96	0.96	0.96	0.90	0.90	0.90	1.20	1.20	1.20
Available phosphorus, %	0.49	0.49	0.49	0.42	0.42	0.42	0.35	0.35	0.35
Sodium, %	0.18	0.18	0.18	0.18	0.18	0.18	0.18	0.18	0.18
Fatty acids									
∑ n-3, %	0.08	0.30	0.30	0.06	0.30	0.30	0.05	0.30	0.30
∑ n-6, %	1.45	1.20	1.27	1.20	1.00	1.00	1.14	1.00	1.00
∑ n-6: ∑ n-3 ratio	18.1	4.00	4.23	20.0	3.33	3.33	22.8	3.33	3.33

1 Starter: d 0–4; Grower: d 5–19; and, Prebreeder: d 20–22.

2 Microalgae (*Aurantiochytrium limacinum*) fermentation product (DMA), as a source of docosahexaenoic acid, Alltech Canada, Guelph, Ontario, Canada.

3 Co-extruded full-fat flaxseed and pulse mixture (FFF, 1:1 wt/wt), as a source of α-linolenic acid, O & T Farms Ltd., Saskatoon, Saskatchewan, Canada.

4 Provided in kg of diet: vitamin A (retinol), 10,000 IU; vitamin D3 (cholecalciferol), 3,000 IU; vitamin E, 100 mg; vitamin K3 (menadione), 5.0 mg; vitamin B1 (thiamin), 4.0 mg; vitamin B2 (riboflavin), 10.0 mg; vitamin B3 (niacin), 50.0 mg; vitamin B5 (pantothenic acid), 20.0 mg; vitamin B6 (pyridoxine), 4.0 mg; vitamin B9 (folic acid), 2.0 mg; vitamin B12 (cyanocobalamin), 30.0 mg; biotin, 200 mcg; choline, 400.0 mg; Mg, 110 mg; Zn, 80 mg; Fe, 40.0 mg; Cu, 10.0 mg; I, 1 mg, Se, 0.31 mg.

5 Provided 2,800 U of cellulase, 400 U of mannanase, 50 U of galactanase, 1,000 U of xylanase, 600 U of glucanase, 2,500 U of amylase, and 200 U of protease per kilogram of diet. (Superzyme OM; Canadian Bio-Systems Inc., Calgary, Alberta, Canada)

6 SANTOQUIN, Novus International Inc., Saint Charles, MO.

### Experimental Procedures and Sampling

The diets were allocated based on pen body weight (**BW**) to nine replicate pens for the control and 6 replicates each for DMA and FFF diets. The rationale for allocating 3 extra replicates to the control group was because this study was part of a larger project, set up in a factorial design that followed pullets into laying phase performance (data not shown). Briefly, the laying phase required birds reared on control diets to be split further to continue on the control diet or start on either FFF or DMA diets and as such more birds were placed on control during rearing. Birds were fed ad libitum during the first week of life. Subsequently, feed allotment was adjusted weekly based on pen population and average body weight (**BW**) according to breeder guidelines (Aviagen, 2013). The birds were on a daily feeding schedule. Birds had ad libitum access to water. From 2 woa and onward birds were individually weighed on a weekly basis for calculation BW uniformity, BW gain (**BWG**), and feed conversation ratio (**FCR**). Six birds per pen were randomly euthanized via cervical dislocation at 5 and 12 woa and dissected for empty gizzard, empty small intestine, ceca, spleen, liver, and bursa weight. Two mid-jejunum segments per bird per pen (2.5 cm) were placed in 10% formalin for histomorphology analyses.

### Sample Processing and Analyses

Experimental diets were finely ground in a coffee grinder and thoroughly mixed. Samples were analyzed for dry matter (**DM**), crude protein (**CP**), crude fat, starch, calcium, and phosphorous in a commercial laboratory (SGS Canada Inc, Guelph, ON, Canada). Gross energy was determined using a bomb calorimeter (IKA Calorimeter System C 6000; IKA Works, Wilmington, NC). Fatty acid concentration in feed samples was determined in a commercial lab (Activation Laboratories, Ancaster, ON, Canada) according to O'fallon et al. (2007). Fixed jejunal tissue samples were cut into longitudinal cross-sections and embedded in paraffin wax (AHL Laboratories, Guelph, ON, Canada). The tissues were then sectioned and stained with hematoxylin and eosin for morphological measurements (Lu et al., 2019). Villus height (**VH**) and crypt depth (**CD**) were measured (μm) with a calibrated micrometer for each tissue using a Leica DMR microscope at 8 different points (Leica Microsystems, Wetzlay, Germany). The VH: CD ration was calculated by dividing VH by CD.

### Calculations and Statistical Analysis

The BW uniformity was presented as coefficient of variation (**CV, %**) calculated by dividing BW standard deviation by the average pen BW. Organ weights were expressed relative to BW (mg/g BW) for statistical analyses. Statistical analysis was conducted using PROC GLIMMIX procedures of SAS 9.4. The model for BW, BWG, FCR, and organ weights had fixed effects of diet and analyzed by phase or sampling age. The model for BW C.V. had fixed effects of diets and age and the interaction between age and diet. Differences were considered significant at *P* ≤ 0.05.

## RESULTS

The analyzed chemical composition of the experimental diets is shown in Table 2. Supplementation with DMA and FFF changed dietary FA profile. The DMA increased DHA (C22:6) concentration and FFF increased ALA (C18:3) concentration. The concentration of FA in pre-breeder diets was lower than the calculated values. Diets supplemented with omega-3 FA sources exhibited lower n-6: n-3 ratio in all phases.

**Table 2:** Analyzed composition of experimental diets on an as fed basis^1^.

Item	Starter			Grower			Pre-breeder		
	Control	DMA^2^	FFF^3^	Control	DMA	FFF	Control	DMA	FFF
Dry matter, %	87.3	85.5	86.23	87.0	86.9	87.3	87.4	87.4	87.1
Crude protein, %	18.4	19.3	19.4	16.9	16.9	16.9	16.0	16.3	16.9
Gross energy, kcal/kg	3,417	3,425	3,411	3,818	3,827	3,845	3,831	3,822	3,770
Crude fat, %	2.63	2.74	2.49	2.56	2.42	2.45	2.93	3.25	2.86
Starch, %	42.4	43.6	43.0	36.0	38.0	37.3	38.1	36.0	40.4
Calcium, %	0.93	0.96	0.84	0.78	0.68	0.78	0.79	1.33	1.41
Phosphorus, %	0.60	0.76	0.69	0.78	0.69	0.80	0.65	0.66	0.70
Fatty acids, g/100 g									
C18:3	0.097	0.082	0.259	0.092	0.079	0.316	0.034	0.030	0.113
C 20:5	<0.001	0.001	<0.001	<0.001	0.002	<0.001	0.001	0.002	0.001
C 22: 6	<0.001	0.094	0.017	<0.001	0.149	0.019	<0.001	0.053	0.023
∑ n-3, %	0.093	0.170	0.264	0.088	0.221	0.321	0.037	0.087	0.119
∑ n-6, %	1.470	1.200	1.210	1.460	1.320	1.280	0.552	0.466	0.488
∑ n-6: ∑ n-3 ratio	15.81	7.06	4.58	16.59	5.97	3.99	14.75	5.33	4.09

1 Starter: d 0–4; Grower: d 5–19; and Prebreeder: d 20–22.

2 Microalgae (*Aurantiochytrium limacinum*) fermentation product (DMA), as a source of docosahexaenoic acid, Alltech Canada, Guelph, Ontario, Canada.

3 Co-extruded full-fat flaxseed and pulse mixture (FFF, 1:1 wt/wt), as a source of α-linolenic acid, O & T Farms Ltd., Saskatoon, Saskatchewan, Canada.

**Figure 1:**
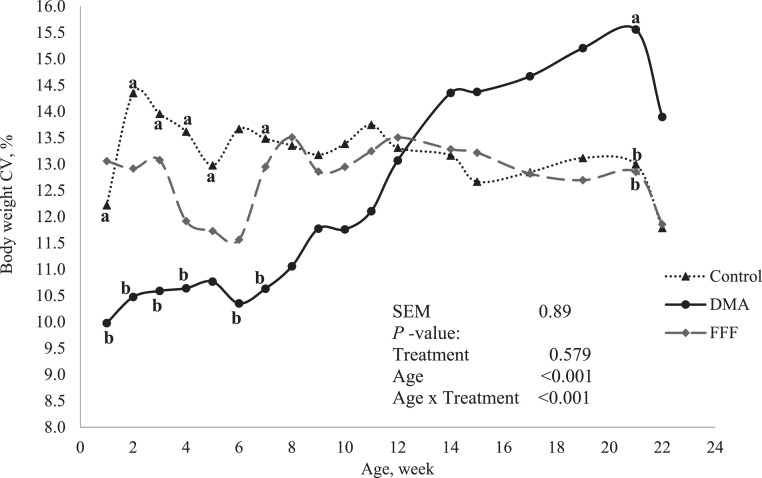
Effects of feed sources of mega-3 fatty acids^1^ on coefficient of variation of body weight at different time points through to wk 22 of age in broiler breeder pullets^2^. ^1^Microalgae (*Aurantiochytrium limacinum*) fermentation product (DMA), as a source of docosahexaenoic acid, Alltech Canada, Guelph, Ontario, Canada. Co-extruded full-fat flaxseed and pulse mixture (FFF, 1:1 wt/w t), as a source of α-linolenic acid, O & T Farms Ltd., Saskatoon, Saskatchewan, Canada. ^2^Different letters represent a significant difference (*P* < 0.05) between treatment groups at a given week of age (age). BW C.V. % was intermediate in FFF fed group from wk 0–8.

There was no diet effect (*P* < 0.10) of diet on BW, BWG, and FCR in the starter and grower phases (Table 3). During the pre-breeder phase there were no dietary effects on final BW or FCR, however, birds fed DMA diets had the highest BWG relative to birds fed control or FFF diets (*P* = 0.049). There was an interaction (*P* < 0.001) between age and diet on BW CV (Figure 1). The BW CV in DMA fed pullets was lower than for pullets fed control between 2 and 7 woa with exception of 5 woa. However, pullets fed DMA had higher BW CV at 20 woa than birds fed control of FFF. The pullet BW CV was similar (*P* > 0.05) among diets at 22 woa.

**Table 3:** Effects of feeding sources of omega-3 fatty acids on growth performance in broiler breeder pullets from hatch through to 22 wk of age.

Item	Control	DMA^1^	FFF^2^	SEM	*P-*value
Starter, wk 0–4					
Body weight (BW), g/bird	430	446	433	5.44	0.119
BW gain, g/bird	317	330	321	6.26	0.326
FCR	2.49	2.42	2.49	0.05	0.460
Grower, wk 5–19					
Body weight (BW), g/bird	1,837	1,823	1,829	15.60	0.808
BW gain, g/bird	1,388	1,377	1,396	15.16	0.716
FCR	4.12	4.15	4.09	0.19	0.706
Pre-breeder, wk 20–22					
Body weight (BW), g/bird	2,053	2,089	2,061	22.82	0.545
BW gain, g/bird	229^b^	268^a^	232^b^	11.85	0.049
FCR	8.26	7.25	8.36	0.19	0.075

1 Microalgae (*Aurantiochytrium limacinum*) fermentation product (DMA), as a source of docosahexaenoic acid, Alltech Canada, Guelph, Ontario, Canada.

2 Co-extruded full-fat flaxseed and pulse mixture (FFF, 1:1 wt/wt), as a source of α-linolenic acid, O & T Farms Ltd., Saskatoon, Saskatchewan, Canada.

a,b Values with uncommon superscripts within each column are significantly different (*P* < 0.05).

There were no diet effects (*P >* 0.05) on small intestine, ceca, gizzard, liver, bursa, or spleen weight at 5 woa (Table 4). At 12 woa, control birds had heavier ceca (*P* = 0.022) than DMA and FFF birds. There were no dietary effects on the other organs weight at 12 woa. At 5 woa, birds fed DMA had taller VH and deeper CD than birds fed either control or FFF (*P* < 0.05) but VH or CD were similar (*P* > 0.05) between control and FFF pullets. At 12 woa, birds fed FFF had taller VH and VH:CD than birds fed control diet but similar (*P* > 0.05) to birds fed DMA (Table 5). Crypt depth was not significantly different (*P* = 0.662) between dietary treatments at 12 woa.

**Table 4:** Effects of feeding sources of omega-3 fatty acids organ weight in broiler breeder pullets at 5 and 12 wk of age.

Item		Body weight, g	Organ weight, mg/ g BW					
			Gizzard	Small intestine	Ceca	Bursa	Liver	Spleen
Diet	Age, wk							
Control	5	574	33.74	29.02	5.22	1.64	20.40	0.84
DMA^1^	5	658	30.65	30.25	5.21	1.95	20.8	1.06
FFF^2^	5	613	31.45	27.90	6.10	1.91	19.38	1.10
SEM		36.86	1.99	1.16	0.50	0.25	1.01	0.09
*P*-value		0.321	0.544	0.398	0.388	0.667	0.619	0.135
Diet	Age, wk							
Control	12	1,146	28.49	24.65	4.57^a^	1.17	15.70	0.76
DMA^1^	12	1,210	30.11	20.28	3.57^b^	1.62	15.25	0.83
FFF^2^	12	1,204	27.32	20.41	3.68^b^	1.11	15.73	0.77
SEM		60.68	1.35	2.09	0.25	0.216	0.37	0.07
*P*-value		0.719	0.366	0.274	0.022	0.222	0.605	0.733

1 Microalgae (*Aurantiochytrium limacinum*) fermentation product (DMA), as a source of docosahexaenoic acid, Alltech Canada, Guelph, Ontario, Canada.

2 Co-extruded full-fat flaxseed and pulse mixture (FFF, 1:1 wt/wt), as a source of α-linolenic acid, O & T Farms Ltd., Saskatoon, Saskatchewan, Canada.

a,b Values with uncommon superscripts within each column are significantly different (*P* < 0.05).

**Table 5:** Effects of feeding sources of omega-3 fatty acids on jejunal histomorphology in broiler breeder pullets.

Item		Villi height (VH), µm	Crypt depth (CD), µm	VH:CD
Diet	Age, wk			
Control	5	1,246^b^	318^b^	3.91^a^
DMA^1^	5	1,435^a^	379^a^	3.90^a^
FFF^2^	5	1,162^b^	331^b^	3.51^b^
SEM		44.02	10.35	0.12
*P*-value		0.001	0.002	0.025
	Age, wk			
Control	12	869^b^	262	3.40^b^
DMA	12	957^a^	271	3.58^ab^
FFF	12	986^a^	265	3.82^a^
SEM		21.8	7.14	0.11
*P*-value		0.005	0.662	0.019

1 Microalgae (*Aurantiochytrium limacinum*) fermentation product (DMA), as a source of docosahexaenoic acid, Alltech Canada, Guelph, Ontario, Canada.

2 Co-extruded full-fat flaxseed and pulse mixture (FFF, 1:1 wt/wt), as a source of α-linolenic acid, O & T Farms Ltd., Saskatoon, Saskatchewan, Canada.

a,b Values with uncommon superscripts within each column are significantly different (*P* < 0.05).

## DISCUSSION

Inclusion of DMA and FFF resulted in lower n-6: n-3 ratio extending previous observations in our laboratory that indicated inclusion levels used in the present study changed FA profiles in poultry diets (Akbari Moghaddam Kakhki et al., 2020a,b). Less FA concentration was assayed in pre-breeder diets, it is difficult to ascribe the basis of these observations given that the crude fat concentration was close to the calculated values. Broiler breeders possess the genetic potential for rapid growth rate and are, therefore, restricted fed starting from the pullet phase to minimize reproductive issues caused by obesity (Heck et al., 2004). In the current study, pullets met breeder guidelines for BW at the end of each feeding phase regardless of the diet fed. While the effects of n-3 FA enrichment during rearing period have not been investigated fully, results of our study concur with report by Mellouk et al. (2018) where there were no differences between the BW of BB fed fish oil and control. Although not confirmed in the present study, the increased body weight gain in DMA fed birds in the pre-breeder phase may be attributed to effects of DMA components on nutrients utilization and metabolism (Fries-Craft et al., 2021).

Bodyweight uniformity in BB flock is an important indicator of proper management (Hudson et al., 2001). During rearing, a highly uniform flock, interpreted as a low BW CV, results in the harmonized onset of sexual maturity, egg production, and more accurate management practices (Pishnamazi et al., 2008). The BW CV observed in the present study varied between 10 and 15.5% and was comparable to a range of 10 to 14% indicated in the breeder guide (Aviagen, 2013). A BW CV of >13% was observed in previous BB pullets reared in the same facility as the present study (Arrazola et al., 2019). Larger and more aggressive birds may out-compete smaller or timid birds because of feeding restriction as BB age implying birds may not have equal access to feed (Zuidhof et al., 2015). The digestibility of DMA diets may have influenced the better BW uniformity relative to the control fed BB early in life, as seen by the increased villi height. We speculate that the higher BW variability in DMA fed BB between wk 15 and 21 may be attributed to wk 16 intramuscular vaccination against Newcastle-infectious bronchitis, potentially affecting antibody titers and immune response and therefore growth trajectory (Puthpongsiriporn and Scheideler, 2005; Akbari Moghaddam Kakhki et al., 2020c ). Birds fed FFF showed better BW uniformity at 22 woa. Differences on responses to these sources of n-3 FA suggested possibility of impact of other components. For example, the microalgae used in the current study as source of DHA contain several other bioactive components such vitamins, chlorophylls, carotenoids, and phycobiliproteins with immunomodulating properties (Koyande et al., 2019; Fries-Craft et al., 2021).

The primary immune organs in poultry are the thymus, spleen, and bursa (El-Katcha et al., 2014). Due to the role of n-3 FA in immune function and membrane biogenesis, we hypothesized changes in immune organ and gastrointestinal (**GIT**) development. Wang et al. (2000) reported an increase in bursa and spleen weight in 4-wk-old laying hens fed n-3 FA enriched diets but this effect declined from wk 4 onward. However, in a study conducted by Al Khalifa et al. (2012) broilers fed diets with increasing fish oil levels had no significant effect on spleen weight at 47 d. It is difficult to conclusively determine why there were no diet effects on immune organ weights.

The nutrient absorptive capacity of the GIT is dependent on the mucosal surface area available for active and passive permeability properties, which can be indicated by the villi height and crypt depth (Ferrer et al., 2003). As majority of nutrients digestion and absorption mainly occurs in the jejunum, jejunal histomorphology is a good indicator of digestive and absorptive capacity (Van Leeuwen et al., 2004; Tancharoenrat et al., 2014). Birds fed DMA had higher VH at 5 wk compared to control or FFF; however, at 12 woa FFF had significantly higher VH than control and DMA was intermediate. A similar pattern was noted with CD where deeper crypt was noted with DMA at 5 woa but there was no difference between the groups at 12 woa. One explanation for these observations could be that the maturation of the GIT over time may help with the digestibility of flaxseeds and increase villi height. Previous research reported an increase in villi height in broilers and 51-wk-old laying hens fed diets containing whole flaxseeds (Apperson and Cherian, 2017; Westbrook and Cherian, 2019). As flaxseeds contain high levels of soluble nonstarch polysaccharides, a more mature GIT has enhanced capacity to digest and absorb more of the available nutrients (Leung et al., 2018; Thanabalan et al., 2020). As well, inclusion of the multicarbohydrase, although intended for the wheat ingredients in the diet, may have affected the digestibility of FFF and DMA – specifically in the later phases where dietary wheat inclusion was reduced, allowing the enzyme to act upon other ingredients. Another explanation could be that the nutritional and absorptive needs of a young and more mature bird may change and require different types of n-3 FA. Gut development in response to varying ratios of n-6: n-3 had more pronounced effect in younger birds (Konieczka et al., 2018). Fewer studies have been conducted on the effect of DHA sources and histomorphology. A study with diets incorporating DHA rich enzymatically hydrolyzed scallop visceral protein and scallop visceral protein indicated an increased villi height relative to the fishmeal control in 42-day-old broiler chickens (Xing et al., 2018). It could be postulated that these processed products' digestibility may lead to better digestion and absorption in younger birds, reflected in villi height. However, despite the villi height differences, in the current study, there were no differences between diets and body weight development.

Feeding sources of n-3 FA had no impact on growth or organ development. However, birds fed DMA had better BW uniformity in starter and grower phases linked to enhanced jejunal digestive/absorptive capacity. Birds fed FFF showed higher jejunal villi than control at 12 woa and better BW uniformity at 22 woa. Differences on responses to these sources of n-3 FA suggested possibility of impact of other components. Nonetheless, the data indicated provision of n-3 FA in developing BB pullets impacted body weight uniformity, an essential metric in BB flock management.
